# Polypyrrole-incorporated conductive hyaluronic acid hydrogels

**DOI:** 10.1186/s40824-016-0078-y

**Published:** 2016-09-29

**Authors:** Jongcheol Yang, Goeun Choe, Sumi Yang, Hyerim Jo, Jae Young Lee

**Affiliations:** School of Materials Science and Engineering, Gwangju Institute of Science and Engineering (GIST), Gwangju, 500-712 Republic of Korea

**Keywords:** Polypyrrole, Hyaluronic Acid, Hydrogel, Conductive, Biomaterials

## Abstract

**Background:**

Hydrogels that possess hydrophilic and soft characteristics have been widely used in various biomedical applications, such as tissue engineering scaffolds and drug delivery. Conventional hydrogels are not electrically conductive and thus their electrical communication with biological systems is limited.

**Method:**

To create electrically conductive hydrogels, we fabricated composite hydrogels of hyaluronic acid and polypyrrole. In particular, we synthesized and used pyrrole-hyaluronic acid-conjugates and further chemically polymerized polypyrrole with the conjugates for the production of conductive hydrogels that can display suitable mechanical and structural properties.

**Results:**

Various characterization methods, using a rheometer, a scanning electron microscope, and an electrochemical analyzer, revealed that the PPy/HA hydrogels were soft and conductive with ~ 3 kPa Young’s modulus and ~ 7.3 mS/cm conductivity. Our preliminary in vitro culture studies showed that fibroblasts were well attached and grew on the conductive hydrogels.

**Conclusion:**

These new conductive hydrogels will be greatly beneficial in fields of biomaterials in which electrical properties are important such as tissue engineering scaffolds and prosthetic devices.

## Background

Various types of hydrogels have gained attention as effective biomaterials for a last few decades. Hydrogels are three dimensional insoluble networks of hydrophilic polymer chains and swell in aqueous solutions. They can absorb a lot of water within their matrices. Hydrogels usually exhibit great biocompatibility, porosity, soft mechanical properties and ease in modification. Therefore, they have been extensively employed for various applications, such as tissue engineering scaffolds, tissue augments, and drug delivery vehicles. Although hydrogels have such good characteristics, hydrogels do not generally possess electrical conductivity [1]. Since electrical signals are involved in various biological events, such as tissue regeneration, muscle movement, cell communications, biomaterials that have electrical conductance have been fabricated to modulate cell/tissue responses for various applications, including tissue engineering scaffolds and bio-electrodes [2].

Recently, conductive polymers, such as polypyrrole (PPy), polyaniline, poly(3,4-ethylenedioxythiophene (PEDOT)), polythiophene, have been used as components for biomaterials and their applications where electrical signaling is important [3, 4] because they have good electrical characteristics and softer mechanical properties than metals [5–7]. Electrical signals can be efficiently transferred at the interfaces between cells and conductive substrates. For example, lower potentials can lead to more effective cellular modulation on conductive substrates compared to on non-conductive substrates allowing uses of lower electrical potentials. To take advantage of hydrogels and electrically conductive polymers for uses as biomaterials, electrically conductive hydrogel can be a promising platform. Conductive hydrogels typically consist of polymeric co-networks of structural polymers and electrically conductive polymers [3, 8–12]. The conductive hydrogel scaffolds have potentials to achieve electrical communications between cells and stimulate cellular activity such as differentiation [13].

In our studies, we synthesized novel conductive hydrogels which are composed of pyrrole incorporated hyaluronic acid (HA) and PPy. HA is a non-sulfated glycosaminoglycan which is a major component of extracellular matrix. HA has been extensively utilized for a number of biomaterial applications due to its many advantages, such as biodegradability, biocompatibility, bioresorption, easy modification with many functional groups. It is also known as interaction with CD44^+^ cells such as normal stem cells (e.g., mesenchymal stem cells, neural stem cells, and hematopoietic stem cells) and cancer stem cells [14–22]. PPy is an organic conductive polymer and can be easily synthesized electrochemically or chemically. PPy displays inherent good conductivity, long-term stability, and biocompatibility [23], which have made PPy useful in numerous application such as biosensor, drug delivery system and other biomaterials [24–27]. In this study, covalent bond formation between HA and pyrrole were designed to enhance structural stability and uniformity of hydrogel. HA-pyrrole conjugates were first synthesized and polymerized together with pyrrole monomers to elongate PPy chains inside the composite hydrogels and also to form crosslinks between HA and PPy chains. Pyrrole monomer and oxidant concentrations were varied to produce different conductive hydrogels (i.e., PyHA-PPy). Additionally, fibroblasts were cultured on the produced PyHA-PPy hydrogels and its adhesion and growth were examined.

## Methods

### Materials

1-(2-cyanoethyl) pyrrole, lithium aluminum hydride, N-(3-dimethylaminopropyl)-N’-ethylcarbodiimide hydrochloride (EDC), N-Hydroxysuccinimide (NHS), Ammonium persulfate (APS), and diethyl ether were provided from Sigma-Aldrich (St. Louis, MO, USA). Hyaluronic acid (1 × 10^6^ Da) was kindly provided from the LG Life Science Ltd (South Korea). Dulbecco’s modified Eagle’s medium, fetal bovine serum (FBS), and Dulbecco’s phosphate buffered saline (DPBS) were produced from Hyclone. Penicillin/Streptomycin and trypsin/EDTA were provided from Gibco (Gaithersburg, MD, USA). LIVE/DEAD viability/cytotoxicity kit and CMFDA cell tracker kit were purchased from Life Science Technology.

### Synthesis of N-(3-aminopropyl)pyrrole

N-(3-aminopropyl) pyrrole was synthesized as previously described in the literature [27]. In brief, 0.02 mol 1-2(2-cyanoethyl)pyrrole was dissolved in anhydrous ethyl ether (15 mL). The 1-2(2-cyanoethyl)pyrrole solution was added into a LiAlH_4_ solution (0.05 mol in anhydrous ethyl ether, 150 mL). Then, the mixture was refluxed for 12 h. After cooling, the excess hydride was precipitated to a solid form by the addition of the solutions in sequence of water (1.7 mL), 15 % (w/v) NaOH (1.7 mL), and water (5.1 mL). The precipitations were filtered and the remained solvent was completely evaporated. ^1^H NMR (CDCl_3_) was obtained with this material was obtained. 1.9 (m, 2H, CH_2_-2), 2.75 (t, 2H, CH_2_-3), 4.0 (t, 2H, CH_2_-1), 6.1 (d, 2H, CH-β), 6.65 (d, 2H, CH-α).

### Preparation of pyrrole-hyaluronic acid conjugate (PyHA)

0.1 % (w/v) hyaluronic acid sodium salt (HA, 1 × 10^6^ Da, medical use) solution was prepared by dissolving HA powder in deionized (DI) water. EDC (1 mmol) and NHS (1 mmol) were added in to the HA solution. Synthesized N-(3-aminopropyl)pyrrole 1 mmol was then added into the solution. After perfect dissolution pH of the solution pH was adjusted to 5.5 to enhance the reaction yield. After 20 h reaction in room temperature, the solution was dialyzed using (3.5 kDa MWCO, Spectrum laboratories) in DI water at room temperature for 6 days. The water was exchanged every 12 h for three days. The solution was freeze-dried after filtered with 0.22 μm Bottom Top filter (Corning) and stored at −20 °C until use. PyHA was characterized using ^1^H NMR (D_2_O): 1.95 (s, 3H, C(=O)CH_3_), 6.2 (d, 2H, CH-α-pyrrole), 6.7 (d, 2H, CH-β-carbon). Degree of pyrrole subunit substitution is calculated via ^1^H NMR from the ratio of the relative peak integrations of the pyrrole protons and HA methyl protons as ~20 %.

### Fabrication of the PyHA-PPy hydrogels

Polypyrrole/HA composite (PyHA-PPy) hydrogels were fabricated by polymerizing pyrrole within the pre-prepared PyHA hydrogels. To this end, oxidizing agent (i.e., APS) was added to induce PPy polymerization and crosslink the pyrrole moieties attached onto PyHA backbone. The previously synthesized PyHA was dissolved in DI water to have the final concentration (1.0 w/v%). Concentrations of pyrrole solutions (in DI water) were varied to be 0 mM, 10 mM, 25 mM, 50 mM and 100 mM, respectively. Then, the APS solution was prepared in the ranges from 50 mM to 250 mM of final concentrations. PyHA solution and pyrrole solution was mixed together and placed on ice to reach the solution temperature to 0 °C. The APS solution is added into the solution containing PyHA and pyrrole. Then, the mixed solution is vigorously stirred for 30 s and placed between 2 mm gap for 2 h in room temperature. After a hydrogel was formed, the hydrogel sheet moved into the DPBS and incubated for 3 days by changing the DPBS for every 6 h to remove unreacted residual APS and pyrrole monomers inside the hydrogel.

### Mechanical property measurement

The mechanical property of the fabricated hydrogel was measured using a rheometer (KINEXUS). The hydrogel sheet was punched with 6 mm diameter matching with the geometry. The rheological measurement was taken with frequency sweep measurement from 0.1 Hz to 10 Hz with 0.04 strain. The Young’s modulus was calculated from the obtained shear modulus at 1 Hz using the equation according to the literature.

### Electrical property measurement

The electrical property of the hydrogel was measured using the 4-point probe system with Versastat. Before measurement, the hydrogels were washed with DPBS and dried in the air overnight. The dried hydrogels were swollen in DI water. Linear sweep voltammetry was applied and a bulk resistivity of the hydrogel was calculated as shown below.

$$ p=4.53\times \mathrm{t}\times \frac{\mathrm{V}}{\mathrm{I}} $$

where *ρ* is the bulk resistivity and *t* is the thickness of the substrate. The bulk resistivity could be calculated with the equation above. Next, the conductivity (*σ*) was obtained from 1/*ρ*.

### In vitro fibroblast culture

NIH 3 T3 fibroblasts were maintained in DMEM with 10 % FBS, 1 % anti-anti with a 5 % CO_2_ at 37 °C humidified incubator. The medium was changed every 3 days to fresh medium. They were subculture when their confluency reached to 80 %. Subculture was performed with 0.05 % trypsine-0.53 mM EDTA solution treatment for 5 min and cells were collected by centrifugation at 1200 rpm, 5 min. Cell numbers were counted using a hemocytometer. NIH-3 T3 was seeded as 5 × 10^4^ cells/cm^2^.

For the studies of cell growth on the PyHA-PPy hydrogels, the hydrogels were first washed for a week and punched with 8 mm diameter. And then washed with 70 % of ethanol solution for 30 min and extensively washed with DPBS for 3 days, changing the DPBS every day. The NIH 3 T3 were seeded onto the hydrogels at a cell density of 50,000 cells/cm^2^_._ The culture medium was added after 3 h in order to make the cells adhere onto the hydrogels. The medium was changed every 3 day. Cell viability was measured using the Live/dead viability/cytotoxicity kit according to the protocol provided by the manufacturer. In brief, 5 μL of 2 mM calcein AM and 20 μL of 4 mM EthD-1 per 10 mL solution were used. After 10–15 min staining, the individual samples were washed with DPBS twice. Fixing was performed with 3.74 % paraformaldehyde. Fluorescence images were acquired using a fluorescence microscope (Leica DMI3000B). Live and dead cells were counted as green and red colors, respectively. Live cell numbers were counted from at least 5 randomly taken images.

## Results and discussion

### PyHA-PPy hydrogels fabrication

**Scheme 1:**
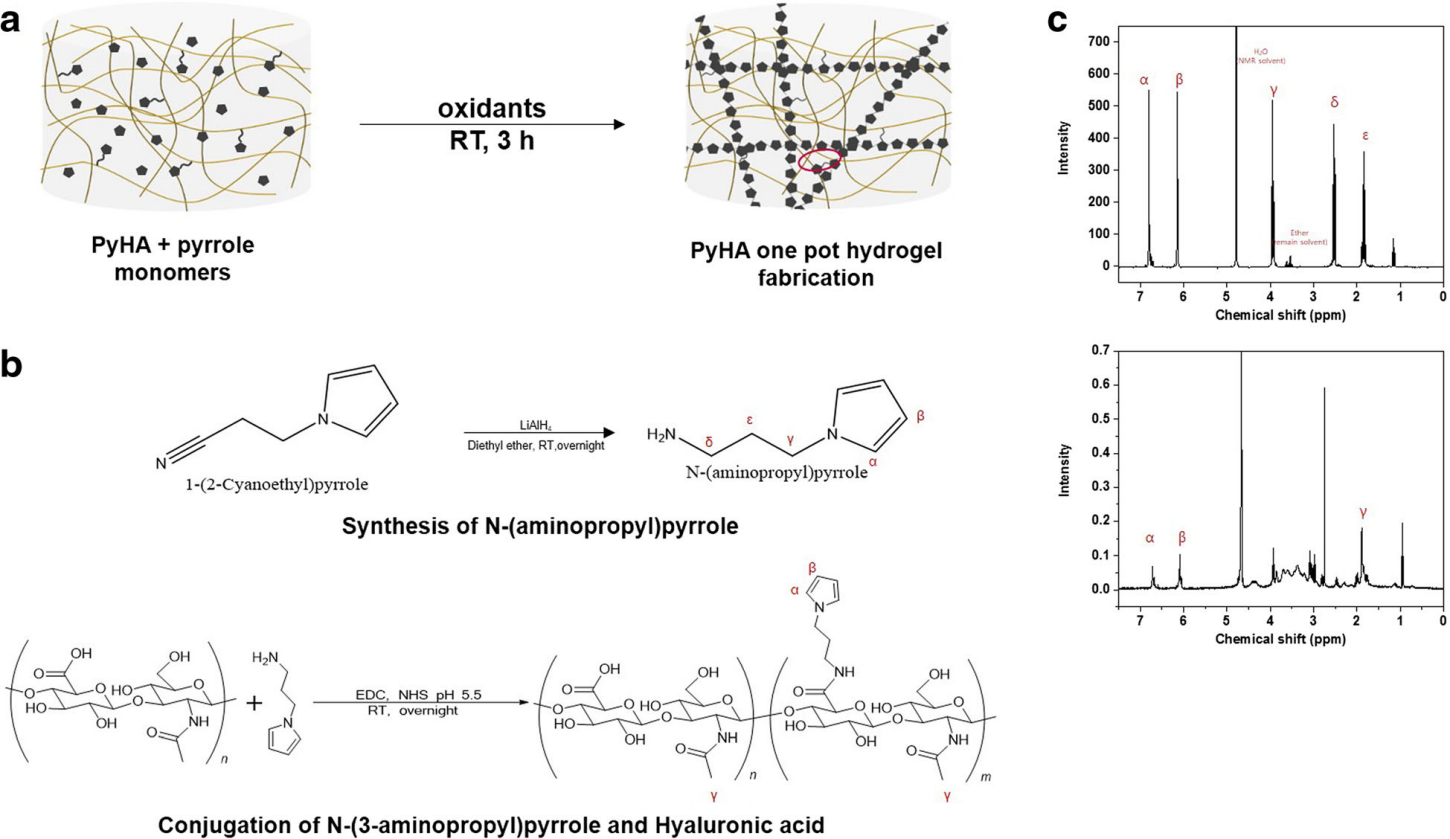
(a) Scheme of PyHA-PPy hydrogels synthesis. (b) Chemistry of N-(3-aminopropyl)pyrrole synthesis and pyrrole-HA conjugate synthesis (c) H^1^ NMR spectra of N-(3-aminopropyl)pyrrole (top) and PyHA conjugate (bottom)

**Fig. 1:**
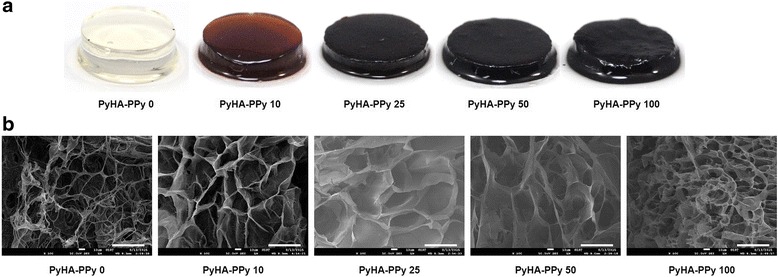
**a** Photographs of the various PyHA-PPy hydrogels. **b** SEM images of the PyHA-PPy hydrogels. Scale bars are 50 μm

The various PyHA-PPy hydrogels were fabricated with the different pyrrole concentrations (i.e., 0 mM, 10 mM, 25 mM, 50 mM, and 100 mM) as shown in Table 1. The fabricated PyHA-PPy hydrogels were clean and not brittle. First, PyHA conjugates were chemically synthesized (Scheme 1). N-(3-aminopropyl) pyrrole was conjugated onto HA backbone using EDC/NHS chemistry. Hydrogel formation was expected to result from the oxidative coupling of the pyrrole moieties between HA chains and/or the coupling between the polymerized PPy chains and the conjugated pyrrole moieties presented on HA. The fabricated hydrogel in this manner could form stable covalent bonds between HA chains and the PPy chains, allowing for its structural stability. The fact that the hydrogel could be formed even without any additional pyrrole monomers in the presence of the APS suggests that the pyrrole moieties on PyHA were associated to form covalent bonds. Furthermore, with an increase in a pyrrole monomer concentration, PPy contents in the PyHA-PPy hydrogels appeared to increase, which could consequently increase stiffness and electrical conductivity. In our studies, as the pyrrole monomer and oxidant concentrations increased, the resultant hydrogels exhibited darker color, which indicates that the added pyrrole monomers were oxidized into PPy with the PyHA hydrogels. As mentioned above, simple mixing of the PyHA solutions and APS without any additional pyrrole monomers could lead to hydrogel formation (Fig. 1a). It should be noted that the sizes of hydrogels decreased after the PPy polymerization with oxidants. These size decreases of the hydrogels were more distinct for the samples synthesized at higher pyrrole monomer concentrations (higher PPy contents). These results may result from the high entanglement degrees due to more chain units and/or decreases in hydrophilicity due to increases in less hydrophilic PPy portions.

**Table 1:** The names of various PyHA-PPy hydrogels and their synthetic conditions

Sample Names	PyHA-PPy 0	PyHA-PPy 10	PyHA-PPy 25	PyHA-PPy 50	PyHA-PPy 100
PyHA solution	1.0 %	1.0 %	1.0 %	1.0 %	1.0 %
Pyrrole	0 mM	10 mM	25 mM	50 mM	100 mM
APS	50 mM	75 mM	100 mM	125 mM	100 mM

All concentrations were described as the final concentrations after mixing all components

### PyHA-PPy hydrogel morphologies

Internal structures of the hydrogels were examined by SEM. All fabricated hydrogels showed the microporous structures inside the hydrogel (Fig. 1b). The pore sizes appeared to be in the ranges of 10 μm. Interestingly, web-like structures with globular shape with size less than 100 nm were observed when the PPy portions were high in the hydrogels. These PPy structures were prominently observed from PyHA-PPy100 hydrogels and PyHA-PPy10 hydrogels. The conventional PPy was reported to have sphere-like structures or web-like globular shape structures when polymerized chemical oxidants. Observed web-like PPy morphologies imply that PPy chains grew inside the hydrogels.

### Characterization of PyHA-PPy hydrogels

**Fig. 2:**
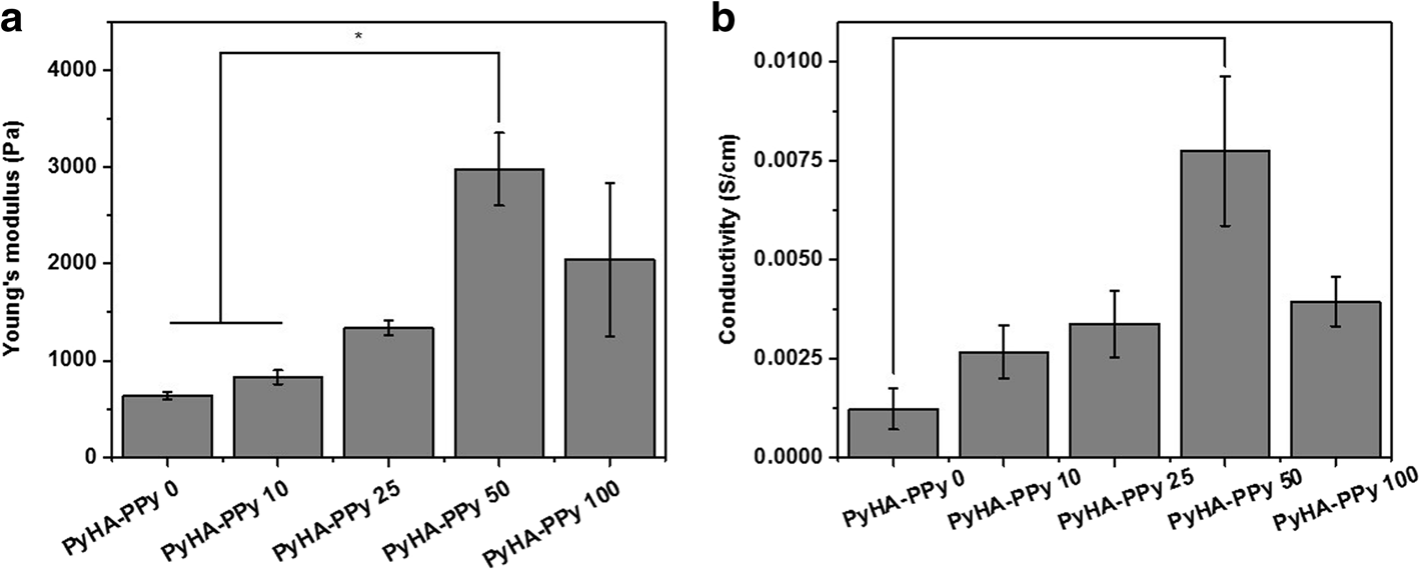
Mechanical and electrical properties of the PyHA-PPy hydrogels. **a** Young’s modulus of the fabricated hydrogels. **b** Conductivity of the fabricated hydrogels. Averages and standard deviations were plotted (*n*=3). Statistical difference was assessed using the student *t*-test and statistical significance was denoted with an asterisk (p < 0.05)

The modulus was measured using an oscillatory rheometer in a frequency sweep mode. The moduli of the hydrogels increased with increases in the added pyrrole monomer concentrations by 50 mM pyrrole. Results indicate that PPy in the hydrogel might contribute the enhancement of the modulus of the hydrogels. The Young’s modulus was in a range from 0.6 kPa to 3 kPa. However, the modulus decreased when the pyrrole concentration was above 50 mM (Fig. 2a). PyHA-PPy 100 hydrogels did not follow the general trend that the increases in the PPy portions inside the hydrogels result in the increases of both the stiffness and electrical properties. It may be due to heterogeneous composite formation by heterogeneous PPy incorporation. Too high pyrrole concentrations and oxidants might lead to too fast reaction rate inside the hydrogel or in the polymerizing solution (outside the hydrogel). Since free pyrrole monomers can be oxidized more readily than the pyrrole moieties attached on PyHA, PPy formed in the solution not in the hydrogels and deposited on the surfaces of the hydrogels. Also, the pyrrole groups on the PyHA might not be sufficiently associated with PPy polymerization in PyHA-PPy 100 hydrogel, resulting in insufficient covalent bond formation in PyHA-PPy and poor stability of mechanical and electrical properties.

The electrical conductivity of the hydrogels was measured (Fig. 2b). There were few reports about the measurement of conductivity of conductive hydrogels and therefore it is difficult to directly compare the results. Hur et al. reported that the PPy agarose based hydrogel showed the conductivity in the order of 10^−1^ S/cm [28]. Shi et al. fabricated the cellulose/polypyrrole composite aerogels and found their conductivity were approximately 0.08 S/cm [26]. Our hydrogels made of PPy and PyHA had the conductivities in a range of 10^−3^ - 10^−2^ S/cm. The conductivity of the hydrogels was increased with the amount of the polypyrrole inside the hydrogel increased until the pyrrole concentration in the polymerizing solution was 50 mM. The highest conductivity, 7.27 mS/cm was measured from the PyHA-PPy 50 hydrogel that also showed the highest stiffness. This observation might be associated with the similar reasons with the weaker mechanical property PyHA-PPy 100 hydrogel than PyHA-PPy 50, which include structural instability and non-uniformity. For example, in the case of the PyHA-PPy 100 hydrogels, too fast reactions led to the local aggregation of PPy on the surface of the hydrogels, which had limited effects on the conductivity.

### In vitro study

**Fig. 3:**
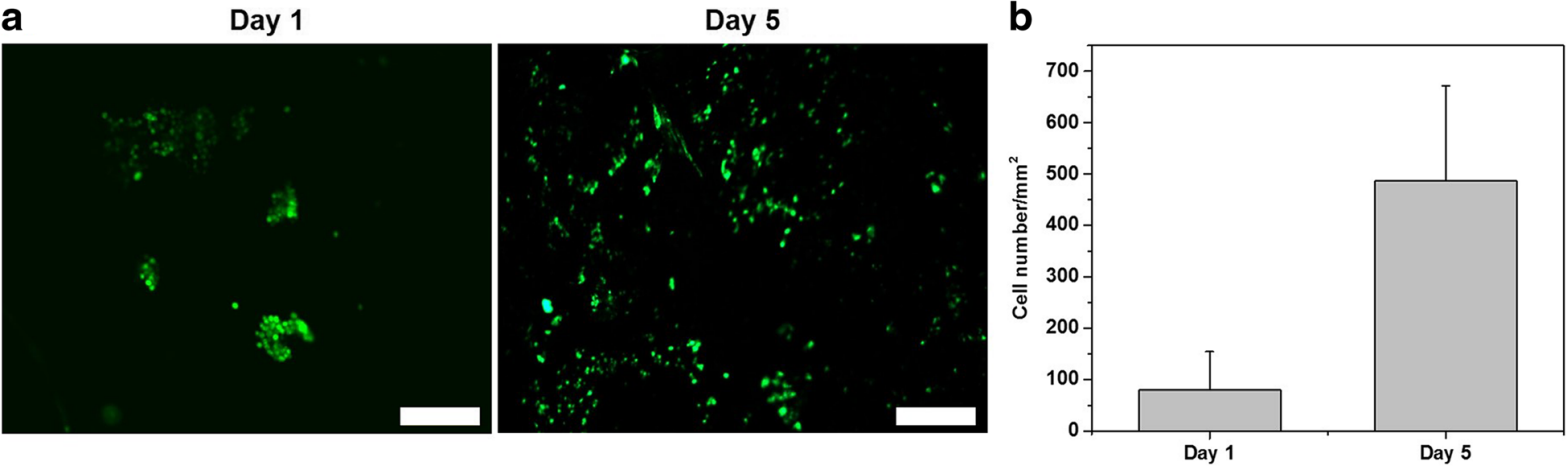
**a** Live/dead staining images of the 3 T3 cells on PyHA-PPy 50 hydrogels at day 1 and 5, respectively. **b** A plot of the attached cell numbers on the PyHA-PPy 50 hydrogel at day 1 and 5. Averages and standard deviations were plotted (*n*=5). Scale bars are 200 μm

Because PyHA-PPy 50 showed the greatest electrical properties with appropriate soft characteristics (~3 kPa Young’s modulus) among differently fabricated PyHA-PPy hydrogels, we selected PyHA-PPy 50 hydrogels for further in vitro studies (Fig. 3). These mechanical and electrical properties of the fabricated PyHA-PPy hydrogels appear to be suitable for the applications where electrical signals were needed in soft environments. We examined the cell adhesion and proliferation on the PyHA-PPy 50 substrates using widely used murine 3 T3 fibroblasts. First, we attempted to culture the cells on the substrates without treatment of any cell-adhesive molecules. Cells were well-attached on the PyHA-PPy 50 mM, indicating the hydrogels’ ability to allow cell adhesion even without any pre-coating. Since HA is generally non-cell adhesive, we speculate that PPy portions might play important roles in promoting cell adhesion. At day 1, about 90 cells/mm^2^ were attached. Adhered cells showed spherical morphology. Further incubation allowed cell proliferation. At day 5, the number of the cells was increased by approximately 6 times (545 cells/mm^2^). In particular, cells showed stretched morphologies, indicating viable cells on the PyHA-PPy. Hence, cells were highly viable on PyHA-PPY 50 mM hydrogel at both days. As results, our PyHA-PPy hydrogels can support cell adhesion and proliferation. Further studies will be needed for the cultivation of other types of cells, such as stem cells and neural cells for specific tissue engineering scaffold applications. Also, studies on the effects of electrical stimulation of cells via our conductive hydrogels will be needed to clearly demonstrate the benefits of conductive hydrogels.

## Conclusion

We fabricated PyHA-PPy hydrogels via covalent bond formation and PPY polymerization. These hydrogels were soft, porous, structurally stable and electrically conductive. As the added pyrrole concentration increased, fabricated hydrogels showed darker color, higher mechanical and electrical properties. Among various conductive hydrogels, the PyHA-PPy 50 showed mM showed the highest 7.3 mS/cm with softness (~3 kPa Young’s modulus). In addition, *in vitro* study showed good cell adhesion and proliferation on the PyHA-PPy 50 substrates. Our new conductive hydrogels will be useful in tissue engineering field which needs electrical stimulation and mechanical softness.

## Abbreviations

APS: Ammonium persulfate
CMFDA: 5-chloromethylfluorescein diacetate
DI: De-ionized
DPBS: Dulbeco’s phosphate buffered saline
FBS: Fetal bovine serum
HA: Hyaluronic acid
PPY: Polypyrrole
PyHA: Pyrrole-conjugated hyaluronic acid
PyHA-PPy: Polypyrrole-incorporated PyHA hydrogel
